# Long-term multimodal imaging in acute posterior multifocal placoid pigment epitheliopathy and association with coxsackievirus exposure

**DOI:** 10.1371/journal.pone.0238080

**Published:** 2020-08-24

**Authors:** Alexa L. Li, Neal V. Palejwala, Jessica G. Shantha, Ghazala O’Keefe, Cecilia S. Lee, Thomas Albini, Steven Yeh

**Affiliations:** 1 Section of Vitreoretinal Disease and Surgery, Department of Ophthalmology, Emory Eye Center, Atlanta, GA, United States of America; 2 Department of Ophthalmology, University of Washington, Seattle, WA, United States of America; 3 Department of Ophthalmology, Bascom Palmer Eye institute, University of Miami Miller School of Medicine, Miami, FL, United States of America; Massachusetts Eye & Ear Infirmary, Harvard Medical School, UNITED STATES

## Abstract

The purpose of this study was to evaluate potential insights into the pathogenesis of acute posterior multifocal placoid pigment epitheliopathy (APMPPE) using multimodal diagnostic imaging and laboratory evaluation in long-term follow-up. A retrospective, single-center case series was conducted on seven consecutive patients (14 eyes) who were given a diagnosis of APMPPE from March 1, 2011, through June 30, 2019 with at least three months of follow-up. Clinical characteristics (age, symptoms, visual acuity [VA]), laboratory testing including coxsackievirus titers, and multimodal imaging from fundus photography, spectral-domain optical coherence tomography (SD-OCT), fundus autofluorescence (FAF), fluorescein angiography (FA), and indocyanine green angiography (ICG) were analyzed for each patient. The initial median VA was 20/71 and final median VA was 20/22. Coxsackievirus B (CVB) titers were elevated (≥ 1:80) in six of seven patients, with a four-fold increase in convalescent titers seen in two patients suggestive of recent infection. All patients were treated with oral corticosteroids, and five patients underwent corticosteroid-sparing immunomodulatory therapy. Initially, multifocal deep choroidal lesions were observed in the posterior pole corresponding to patches of hypocyanescence on ICG. Overlying retinal pigment epithelium (RPE) disease was observed on FAF, although this finding was not universally observed, suggesting that RPE disease may occur as a sequelae to unchecked choroidal inflammation. SD-OCT architectural changes confirmed outer retina and ellipsoid zone disruption. FA of active lesions showed early hypofluorescence and late hyperfluorescence with surrounding leakage while inactive disease showed areas of staining. Long-term follow-up of multimodal diagnostic imaging in APMPPE revealed that choroidal inflammation likely precedes RPE change and photoreceptor damage. Elevation of coxsackievirus titers with seroconversion may be associated with an infectious trigger in concert with immune-mediated disease in this posterior uveitis syndrome.

## Introduction

Acute posterior multifocal placoid pigment epitheliopathy (APMPPE) is a rare cause of sudden painless vision loss in young healthy adults originally described by Gass in a series of three patients in 1968 [1]. The characteristic funduscopic findings of this white dot syndrome include multifocal, flat, cream-colored placoid lesions that are typically bilateral, although asymmetric or sequential presentation is common. A viral prodrome has been reported in up to one-third of cases; however, the precise etiology of this inflammatory condition remains unknown [2]. Viruses such as Coxsackievirus B4 [3] and Adenovirus type 5 [4] have been implicated in association with APMPPE, and vaccinations have also been hypothesized as a potential trigger [5, 6]. Furthermore, HLA-B7 and HLA-DR2 antigens have been linked to APMPPE patients, suggesting an immunological mechanism underlying the pathogenesis of this disease [7]. Although vision loss can be severe and recurrences may also rarely occur, spontaneous and rapid visual recovery is common [8–11]. The benefit of therapy is unclear, as there are no prospective randomized controlled trials. However, corticosteroids or immunosuppressive therapy have recently been advocated to expedite recovery and decrease chorioretinal scarring [12].

Multimodal imaging has been utilized in the evaluation of posterior uveitis syndromes, particularly the white dot syndromes, and may help us to better understand the pathophysiology underlying APMPPE and monitor episodes of inflammation over time. Prior studies have utilized conventional fundus photography, fluorescein angiographic (FA), indocyanine green angiography (ICG-A), optical coherence tomography (OCT), and fundus autofluorescence (FAF) to characterize the disease [13–18]. Goldenberg et al. [13] developed a 4-stage classification system of APMPPE that described spectral-domain OCT findings from disease onset to resolution. Studies utilizing fundus autofluorescence have demonstrated the delayed involvement of RPE and suggest that the initial events that lead to a clinical appearance of APMPPE may be linked to a choroidal vasculitis [14, 15]. There is a lack of literature describing the long-term follow-up of multimodal imaging to characterize the anatomical level of disease activity from onset to resolution in combination with laboratory testing.

While imaging modalities have allowed us to better ascertain the location of inflammation, the etiology and pathogenesis of this rare inflammatory condition has not been clearly elucidated. Coxsackievirus has previously been associated with unilateral acute idiopathic maculopathy (UAIM) and other ocular inflammatory disorders [19–23]. The imaging features in UAIM overlap with characteristics present in APMPPE [24], demonstrating serial changes, which begin within the choroid, and subsequently result in RPE and overlying photoreceptor damage. Based on these similar imaging findings, we hypothesized that coxsackievirus could also play a potential role in triggering an immune-mediated response leading to APMPPE based on these similar choroidal inflammatory processes. In addition, recent studies have shown that the extended spectrum of UAIM may also include bilateral disease[25].

We undertook this laboratory diagnostic study and long-term multimodality imaging review to characterize the pathogenic features of APMPPE and to explore the potential association between coxsackievirus exposure and APMPPE. We describe a series of patients in whom multimodal imaging allowed visualization of the choroidal, RPE and retinal changes, which progressed in patients who showed laboratory findings consistent with the evolution of coxsackievirus exposure from the acute to convalescent phases of disease.

## Materials and methods

The Institutional Review Board of Emory University approved this study, and all work pertaining to this project maintained compliance with the Health Insurance Portability and Accountability Act and adhered to the tenets of the Declaration of Helsinki. We reviewed the medical records of consecutive patients who were given a diagnosis of APMPPE from March 1, 2011, through June 30, 2019 at Emory Eye Center who had at least three months of follow-up. Seven patients with clinical features of APMPPE were included for analysis. The demographic information, age at presentation, visual and systemic symptoms, ocular and medical history, family history, and detailed medication history were recorded for each patient. Best-corrected visual acuity (BCVA), clinical diagnostic imaging (fundus photography, spectral-domain OCT, FAF, FA, and ICG-A), and laboratory testing including coxsackievirus B1-B6 (CVB) antibody titers were also reviewed. Coxsackievirus B antibody testing was performed through semi-quantitative serum neutralization (ARUP Laboratories, Salt Lake City, UT) with a reference interval less than 1:10 [26]. Positive antibody titers greater than or equal to 1:80 were indicative of probable past or current infection, and an increase in antibody titers between acute and convalescent sera of at least four-fold (seroconversion) was considered strong evidence of recent or current infection [26].

Retinal photography of the fundus and fluorescein angiography were performed using a Topcon TRC 50DX retinal camera (Topcon America Corporation, Oakland, NJ) and an ultra-widefield imaging system Optos® California (Optos, Marlborough, MA, USA). Spectral-domain OCT images were taken on the Cirrus-HD OCT4000 instrument (Carl Zeiss Meditec, Inc, Dublin, CA). High-speed ICG angiography and FAF images were recorded using a modified confocal scanning laser ophthalmoscope (model HRA2; Heidelberg Engineering, Carlsbad, CA) and the Optos® California (Optos, Marlborough, MA, USA), respectively.

Descriptive statistics were performed with Microsoft Excel (Version 16.16.20, Seattle, Washington). Visual acuities were converted to the logarithm of minimal angle of resolution (logMAR) for calculation of median visual acuity and paired comparisons between initial and final visual acuity.

## Results

### Patient characteristics

The clinical characteristics of the 7 study patients are included in Table 1 (see S1 and S2 Figs for baseline multimodal imaging for all patients). All patients were healthy except for patient 4 who had a history of mesenteric adenitis, which was thought to have an association with an inflammatory process possibly linked to Coxsackievirus. The median age of presentation was 24 (range 13–75) years. Five of the seven patients were female. All patients presented with acute or subacute, painless vision loss. The median interval between symptom onset and presentation was 42 days (interquartile range [IQR] 88 days) and median follow-up was 668 days (IQR 740 days).

**Table 1:** Clinical characteristics of patients with acute posterior multifocal placoid pigment epitheliopathy.

Pt #/ Sex/ Age	Medical Condition/ Viral Prodrome/ Cerebral Vasculitis	Initial Va (OD; OS); Sx	Final Va (OD; OS); Sx	FA	ICG-A	OCT Initial	OCT Final	FAF Initial	FAF Final	Treatment	CVB Acute titers	CVB Convalescent Titers	CVB Recurrence Titers	Total Follow-up (mo)
1/F/23	No/ No/ No	0.7; 0; central scotoma OD	0.1; 0; asx	Early blockage with staining and late leakage of lesions	Dilated choroidal vasculature; multiple choroidal perfusion defects/hypo-cyanescent lesions	CFT OD-212um OS-253um, disruption of outer retina	CFT OD- 210um OS- 248um, intact outer retina with mild RPE scar OD	Heterogeneous lesions with central HypoAF and surrounding HyperAF	HypoAF lesions	IV cortico-steroids with prolonged oral taper, MMF	B3 1:20; B4 1:160	B3 1:320; B4 1:160	N/A	9
2/F/25	No/ No/ No	0; 0; paracentral scotomas OU	0; 0; asx	N/A	N/A	CFT- OD -210um OS- 214um, disruption of outer retina	CFT OD- 214um OS-218um, partial reorganization of outer retina	Heterogeneous lesions with central HypoAF and surrounding HyperAF	HypoAF lesions	Cyclosporine, Azathioprine, oral cortico-steroids	N/A	N/A	N/A	5
3/F/75	HTN/ No/ No	0.6; 0.3; inferior paracentral scotoma OD	0.18; 0.18; asx	Early blockage with late staining and leakage of lesions	Dilated choroidal vasculature, multiple choroidal perfusion defects/hypo-cyanescent lesions	CFT- OD-194um OS-245um, disruption of outer retina	CFT- OD 211um OS- 243um, partial disruption of outer retina OD and mild ERM OS	HyperAF lesions surrounded by hypoAF ring OD, normal AF OS	HypoAF lesions with few stippled areas of hyperAF OD, normal AF OS	Oral cortico-steroids	N/A	B2 1:320; B4 1:80	N/A	34
4/F/16	Mesenteric Adenitis, Iron Deficiency Anemia/ No/ No	1.3; 0.8; central scotoma OU	1.18; 1; vision stable	Optic disc leakage and early blockage of lesions with late staining and leakage	Multiple choroidal perfusion defects/hypo-cyanescent lesions	CFT OD-273um OS- 284um, disruption of outer retina OD, normal outer retina OS	CFT OD-248um OS- 302um, intact outer retina OD, normal outer retina OS	Heterogeneous lesions with central HypoAF and surrounding HyperAF	HypoAF lesions	Oral cortico-steroids, retisert RSS OU, MMF, cyclosporine, MTX, azathioprine	N/A	N/A	B3 1:80; B5 1:80	85
5/F/25	No/No/No	0.3; 0.5; central scotoma OS	-0.12; -0.12; vision stable	Early blockage of lesions with late staining and leakage	Multiple choroidal perfusion defects/hypo-cyanescent lesions	CFT- OD- 282um OS-332um, disruption in outer retina	CFT- OD- 260um OS-305um, mild disruption in outer retina	N/A	HypoAF lesions OD, hypoAF lesions with few stippled areas of hyperAF OS	Oral cortico-steroids	B2 1:40; B3 1:160	N/A	B2 1:20; B3 1:320	20
6/M/14	No/ Yes/ No	0.88; 1; paracentral scotomas OU	0.18; 0.4; asx	Window defects, staining	Multiple hypo-cyanescent lesions	CFT- OD- 178um OS-234um, disruption of outer retina	CFT- OD- 282um OS-332um, disruption of outer retina	Heterogeneous lesions with central HypoAF and surrounding HyperAF	HypoAF lesions	Oral cortico-steroids, MTX	B3 1:80; B4 > = 1:640	B3 1:20; B4 1:160	B3 1:80; B4 > = 1:640	22
7/M/13	No/No/No	0.88; 0; central scotoma OD	0;0; asx	Early blockage and late staining	Multiple hypo-cyanescent areas	CFT- OD- 224um OS-242um, disruption of outer retina	CFT- OD- 178um OS-234um, partial reorganization of outer retina	Heterogeneous lesions with central HypoAF and surrounding HyperAF OD, hypoAF lesions OS	HypoAF lesions	Oral cortico-steroids, azathioprine, MMF	B3 1:320	B3 1:160	N/A	46

AF–autofluorescence; asx–asymptomatic; CFT–central foveal thickness; CVB–coxsackievirus B; ELM–external limiting membrane; ERM–epiretinal membrane; EZ–ellipsoid zone; F–female; FA; fluorescein angiogram; FAF–fundus autofluorescence; HTN–hypertension; hyperAF–hyperautofluorescence; hypoAF–hypoautofluorescence; ICG-A–indocyanine green angiography; IV–intravenous; M–male; MMF—mycophenolate mofetil; mo–month; MTX–methotrexate; N/A–not applicable; OCT–optical coherence tomography; OD–right eye; OS–left eye; pt–patient; RPE–retinal pigment epithelium; um–microns; Sx–symptoms; asx–asymptomatic Va–visual acuity

No patient described a headache, focal neurological symptoms, or signs of cerebral vasculitis prior to or after the onset of visual symptoms. One patient (patient 6) had a viral prodrome prior to the onset of visual symptoms; no patients presented with acute systemic manifestations of hand-foot-mouth disease or had preceding contact with others with this disease.

The median visual acuity of both eyes at presentation and at the end of follow up was 20/71 (20/20-20/400) and 20/22 (20/20-20/300), respectively. All patients were treated with corticosteroids (1 of 7 patients (14%) received IV corticosteroid, 1 patient (14%) received periocular steroid in both eyes, and 7 patients (100%) received oral corticosteroid), and 5 patients (71%) received immunomodulatory therapy (mycophenolate mofetil (43%), oral cyclosporine (29%), oral azathioprine (43%), and subcutaneous injection of methotrexate (29%)).

### Coxsackievirus titers

Coxsackievirus B (CVB) titers were elevated to a value of at least ≥ 1:80 in all 6 of the 6 patients in whom the coxsackievirus titers were checked, indicating prior or current infection. Patient 2 did not have coxsackievirus titers drawn. Coxsackievirus B2 (CVB2) titers were elevated in two patients (33%), coxsackievirus B3 (CVB3) titers in five patients (83%), coxsackievirus B4 (CVB4) titers in three patients (50%), and coxsackievirus B5 (CVB5) titers in one patient (17%) (Table 1). Five of the 6 patients (83%) had elevated titers of multiple CVB serotypes.

Notably, the convalescent coxsackievirus antibody titers demonstrated a greater than four-fold increase compared to antibody titers during the acute phase of disease in 2 of the 6 patients, suggesting recent or active coxsackievirus infection. For patient 1, CVB3 titers were 1:20 and CVB4 titers were 1:160 during the acute phase of inflammation. The patient received intravenous corticosteroids and a prednisone taper, and one month later, convalescent coxsackievirus titers demonstrated a greater than four-fold increase in CVB3 titer (1:320) and stable CVB4 (1:160) titer.

Similarly, patient 6 initially presented with elevated CVB3 titers of 1:80 and CVB4 titers of ≥1:640 in the acute phase of inflammation. High-dose prednisone was initiated, and at a subsequent visit four months later, CVB3 titers decreased to 1:20 and CVB4 titers to 1:160 with the exam showing inactive disease. Approximately one year after the initial visit, the patient presented with a recurrence of new chorioretinal lesions, which corresponded with a four-fold increase in CVB3 titers of 1:80 and a similar four-fold increase in CVB4 titers of ≥1:640, an immunologic response suggestive of a recent re-exposure to coxsackievirus B. The patient was eventually started on methotrexate and the lesions resolved with residual pigmentary changes. At the final follow-up 7 months later, CVB3 titers decreased to 1:20 and CVB4 titers to 1:320.

### Multimodality imaging features

#### Funduscopy

**Fig 1:**
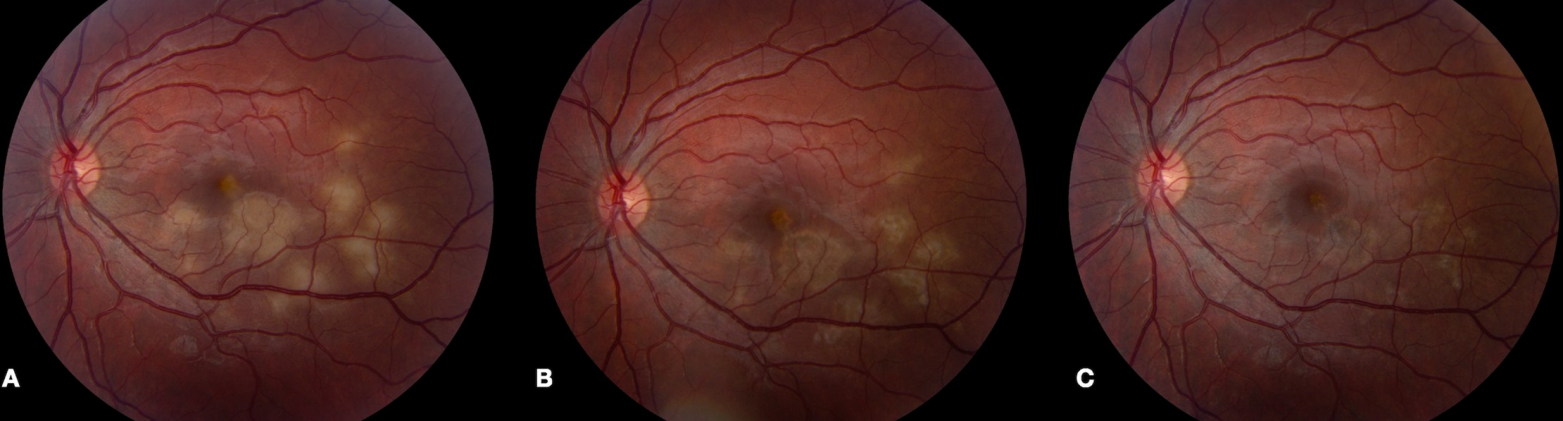
Fundus changes in APMPPE during active and inactive stages of disease. (A) Fundus photo of the left eye of patient 5 demonstrates multiple cream-colored placoid parafoveal lesions indicative of active disease. (B) Five days after treatment with high-dose prednisone, the lesions decreased in size with the interval development of overlying RPE hyperpigmentation. (C) One month later, fundus photo of the left eye revealed RPE pigmentary changes with complete resolution of the previously noted cream-colored lesions.

**Fig 2:**
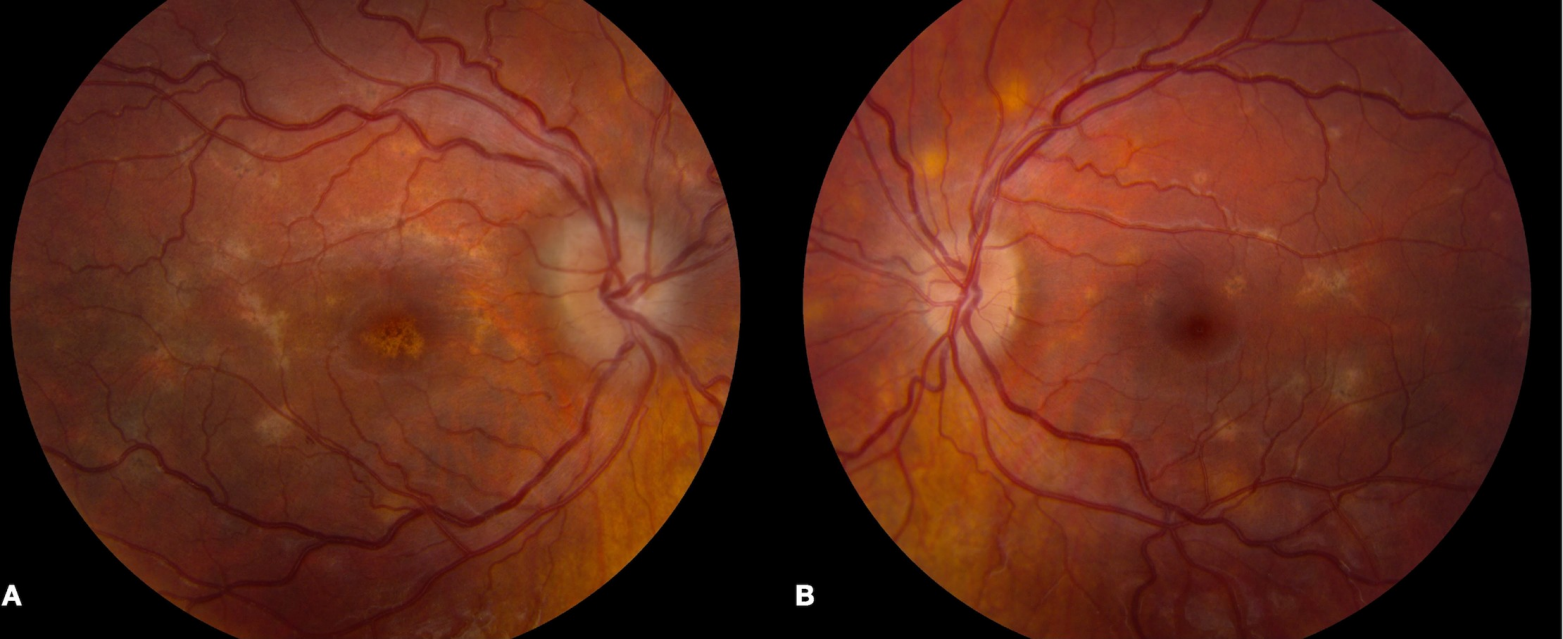
Bilateral disc edema and characteristic fundus findings in patient 4. Fundus photos of patient 4 demonstrated bilateral disc edema and multiple cream-colored lesions in the posterior pole of the right (A) and left (B) eyes.

During the acute phase of inflammation, all patients presented with multifocal deep retinal/choroidal lesions in the posterior pole. The lesions were creamy-white and flat without any overlying exudative neurosensory retinal detachment. The degree of outer retinal and retinal pigment epithelial change varied between patients depending on the stage of the disease indicative of its presentation in various stages of healing (Fig 1). Lesions responded to immunosuppressive therapy, with pigmentation of the borders of the lesion and fading of the cream-colored lesions. Signs of disease inactivity included well-demarcated borders, RPE atrophy with pigmentary changes at the level of the RPE, and the absence of further cream-colored active lesions (Fig 1). Patient 4 presented with optic nerve head edema bilaterally in addition to the characteristic lesions described above (Fig 2).

### Indocyanine green angiography

**Fig 3:**
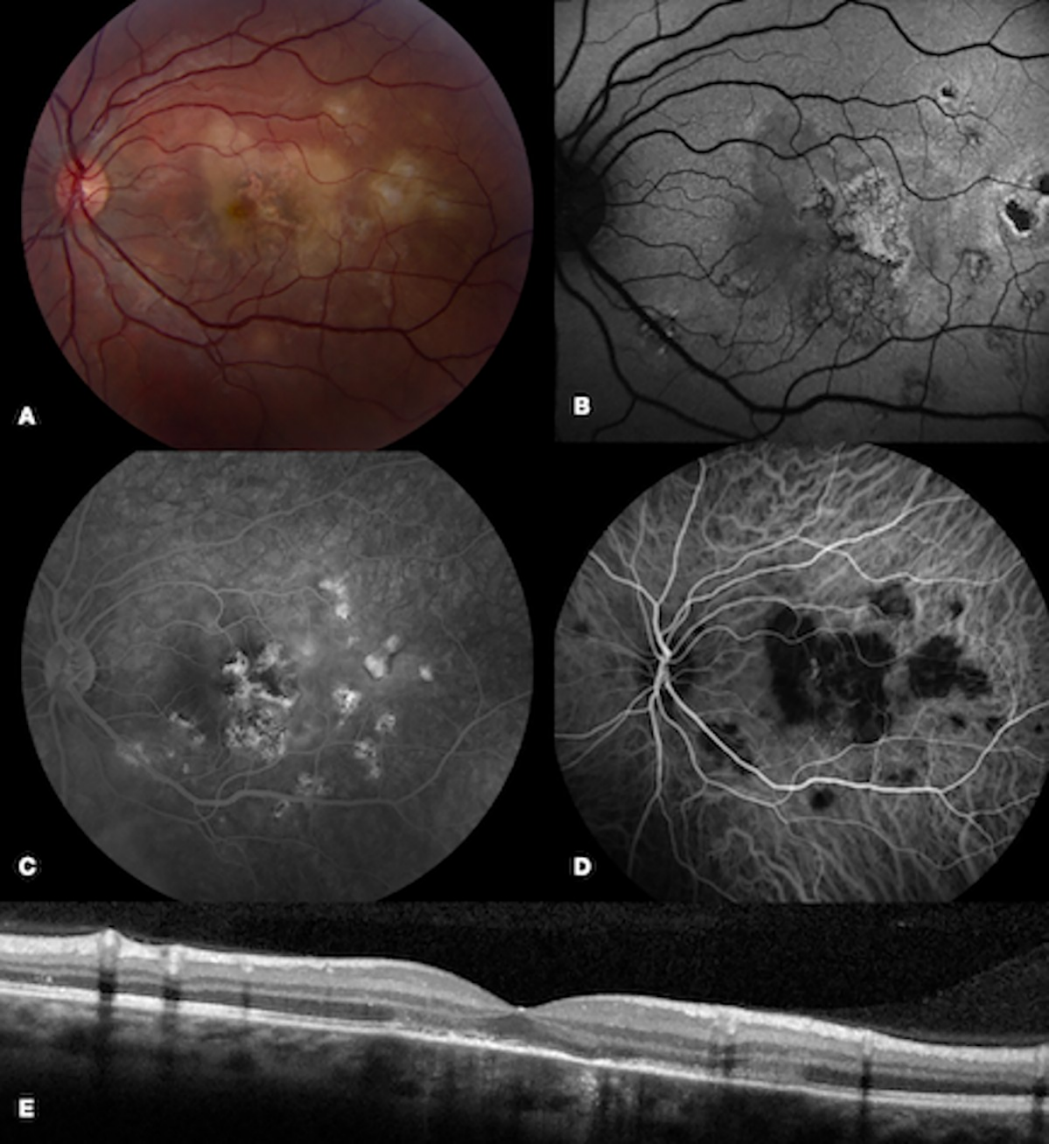
Multimodal imaging during a recurrence of inflammation in patient 5. Fundus photos of the left eye in patient 5 demonstrated multiple new cream-colored lesions near the fovea (A). FAF (B) showed stippled mixed hyperautofluorescent and hypoautofluorescent lesions and FA (C) revealed late hyperfluorescence. ICG (D) demonstrated hypocyanescent lesions that corresponded to the clinically visible lesions. OCT (E) displayed areas of IS-OS disruption.

**Fig 4:**
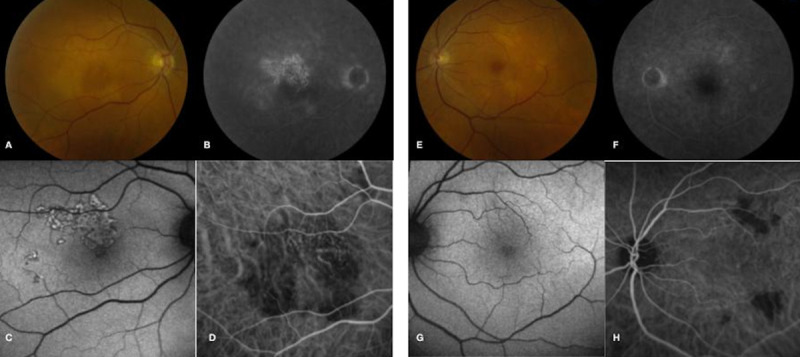
Multimodal imaging demonstrates lesions are more apparent on ICG-A than other imaging modalities in patient 3. Initial fundus photograph of the right eye (A) shows multifocal cream-colored lesions with leakage on FA (B). FAF (C) shows hyperautofluorescence only partially corresponding to the area of choroidal disease, while ICG-A (D) outlines hypocyanescent lesions that appear larger than visualized on fundus photography or FAF. In the left eye, a fundus photograph (E) shows choroidal lesions, minimal FA hyperfluorescence (F) and minimal changes on FAF (G). ICG-A (H) highlights the choroidal lesions that are more numerous than seen on fundus exam or FAF.

Six of the 7 patients underwent ICG-A. Dilated choroidal vascular channels and areas of hypocyanescence were observed, and corresponded to the lesions seen on fundus exam (Fig 3). Interestingly, the lesions on ICG-A appear larger and more numerous than those seen on fundus exam or on any other imaging modality reviewed (Fig 4). In patient 1, ICG-A was obtained within 3 weeks from the onset of symptoms. Lesions demonstrated a scalloped border with more acute lesions displaying a homogenous pattern of hypocyanescence and chronic lesions demonstrating a heterogenous pattern with central speckled hypercyanescence.

### Fluorescein angiography

Six out of 7 patients underwent FA. All demonstrated early hypofluorescence followed by late hyperfluorescence. The hypofluorescence was consistent with a blocking pattern, while the hyperfluorescence was more consistent with leakage in acute phases and with staining in disease quiescence. Patient 4 also showed disc leakage consistent with disc edema on FA.

### Spectral-domain optical coherence tomography

**Fig 5:**
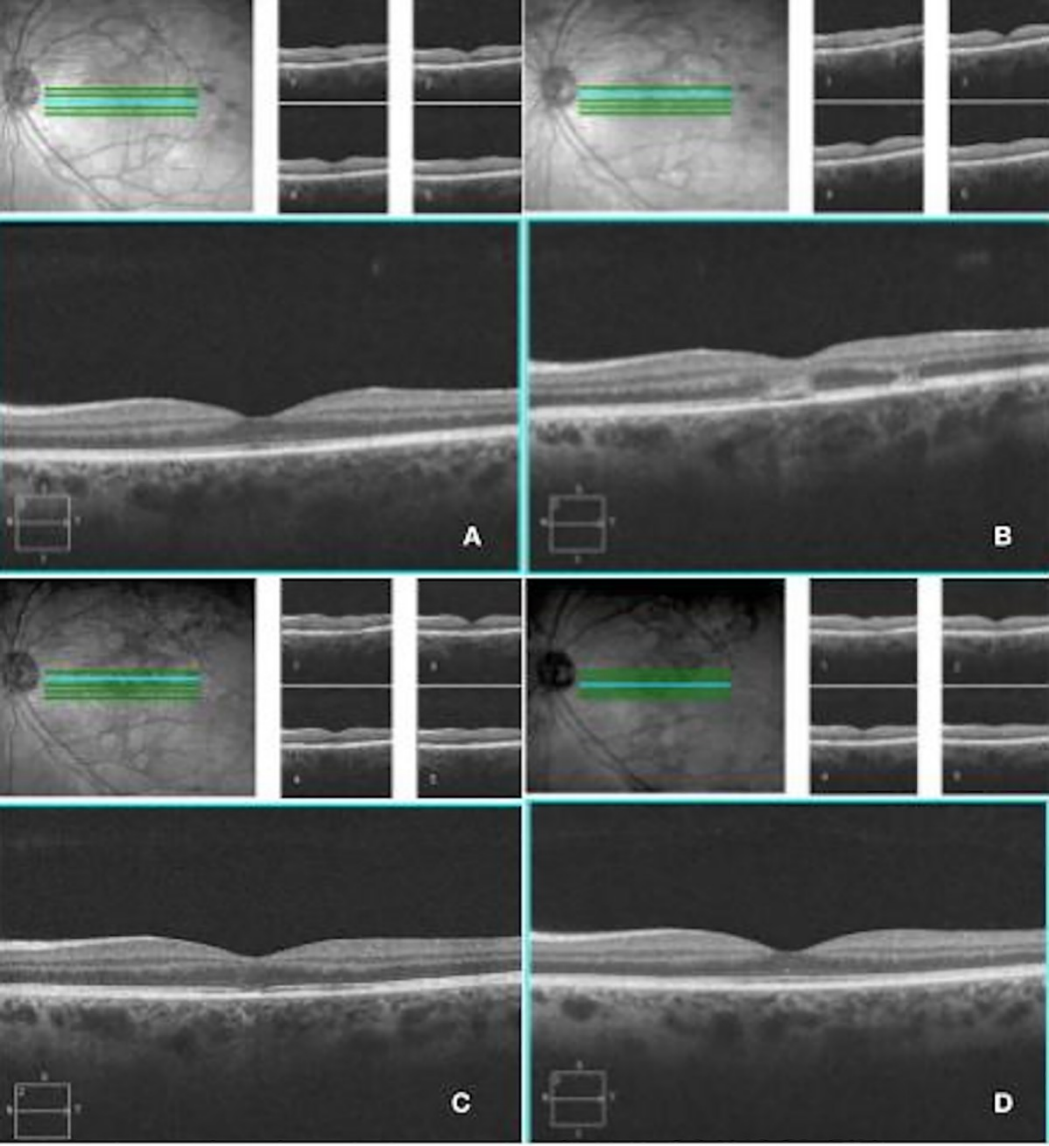
OCT findings from disease onset to resolution in Patient 1. A) Before any retinal/RPE changes are seen, OCT shows thickened choroid with dilated vessels. B) As fundus lesions appear, disruption in the RPE and outer retinal structures can be identified. C) As inflammation subsides, there is partial regeneration in the ellipsoid zone which corresponds to visual recovery. D) At disease resolution, there is complete restoration of the RPE and outer retinal integrity with concomitant clinical improvement.

Spectral-domain OCT was performed in all patients. During the acute disease of illness, disruption of the outer retinal architecture was evident. Irregularities were seen in the external limiting membrane (ELM), ellipsoid zone, and in the RPE (Fig 3). There was also evidence of focal choroidal thickening in the region of the lesion. Of note, no patients had any intraretinal or subretinal fluid on OCT exam. With resolution of active disease, re-organization of the outer retina can be observed on OCT (Fig 5). The ELM and ellipsoid zone appeared more well-delineated; however, focal loss of the RPE layer as well as retinal thinning/atrophy were observed in some areas, particularly within the outer retina.

### Fundus autofluorescence imaging

**Fig 6:**
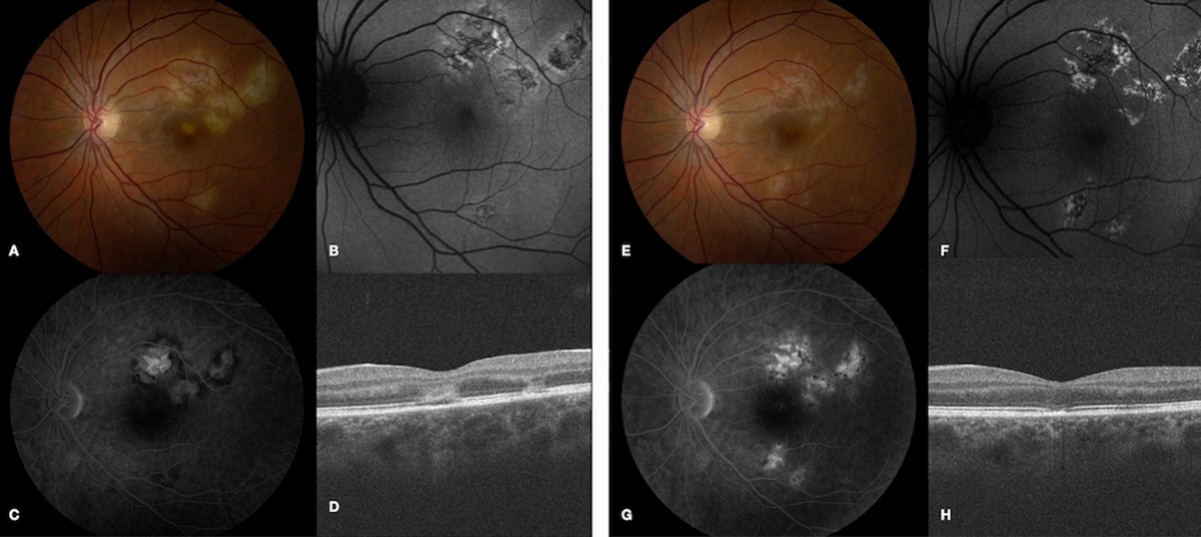
Multimodal imaging during active and inactive disease in patient 1. (A) Fundus photograph in patient 1 during active disease shows fovea-threatening cream-colored placoid lesions. (B) FAF reveals a heterogeneous pattern of central hypoautofluorescent lesions with surrounding stippled hyperautofluorescence. (C) Late stage FA demonstrates late leakage of the placoid lesions without foveal involvement. (D) OCT shows outer retinal architectural disruption. (E) Fundus photography one month later reveals RPE hyperpigmentation and resolution of cream-colored lesions. (F) FAF at this time point demonstrates hypoautofluorescent lesions with surrounding stippled hyperautofluorescence. (G) Late stage FA shows staining with resolved areas of early blockage and late leakage. (H) OCT demonstrates partial restoration of the ellipsoid zone.

All patients underwent FAF imaging. The alteration in autofluorescence corresponded to the clinically significant lesion on funduscopic examination. Interestingly, however, RPE alteration was not universally observed over deep retinal/choroidal lesions, suggesting that the choroidal disease, if treated effectively, could be halted and avert damage to overlying RPE and photoreceptors. Early in the course of disease, discrete lesions with a complex, mixed pattern of hypoautofluorescence and hyperautofluorescence were observed (Fig 6). Lesions typically consisted of a central zone of hypoautofluorescent with a surrounding stippled border of hyperautofluorescence. Later in the disease course, lesions appeared more confluent with indistinct boundaries and a more homogenous hypoautofluorescent pattern suggesting loss of RPE integrity.

### Report of selected cases

#### Patient 1

A 23-year old healthy female was referred for the evaluation of a 5-day history of a central scotoma of the right eye without a viral prodrome. On initial evaluation, BCVA was 20/100 in the right eye and 20/20 in the left eye. Funduscopic examination revealed a central fovea-involving choroidal opacity in the right eye and several parafoveal cream-colored deep choroidal lesions in both eyes. ICG revealed multiple hypocyanescent lesions indicative of choroidal perfusion defects. She was diagnosed with APMPPE given the above clinical findings and initiated on oral prednisone 80 mg daily with close follow-up.

She was seen two days later with stable BCVA but with subjective reports of an increasing pinpoint scotoma in the left eye. Funduscopic examination in the left eye revealed concern for an enlarging cream-colored placoid lesion threatening the fovea (Fig 6). Due to concern for progression in the left eye, the patient was admitted for intravenous (IV) corticosteroid treatment. Coxsackievirus titers at this time point revealed a CVB3 titer of 1:20 and an elevated CVB4 titer of 1:160.

After receiving 3 doses of IV solumedrol 1 gram daily, BCVA improved to 20/40 in the right eye and 20/20 in the left eye. Fundus photography showed a decrease in the size of the previously noted cream-colored lesions with an increase in border hyperpigmentation. She was transitioned to a slow taper of oral prednisone. Examination one month later while on the prednisone taper revealed BCVA of 20/20 in both eyes with areas of RPE atrophy in both eyes. FAF demonstrated hypoautofluorescent lesions with decreased intensity of surrounding hyperautofluorescence. Convalescent CVB titers at this visit showed a greater than four-fold increase in CVB3 titers of 1:320 suggestive of a recent infection and stable CVB4 titers of 1:160. The patient was later transitioned to mycophenolate mofetil 1 gram twice daily for the next 7 months without any evidence of disease recurrence.

#### Patient 3

A 75-year old healthy female presented with a 2-week history of a paracentral scotoma in the right eye without symptoms in the left eye. On initial examination, funduscopic examination showed cream-colored deep lesions in the posterior pole in the right eye and several plaque-like deep choroidal lesions in the left eye. FAF demonstrated hyperautofluorescent lesions surrounded by a hypoautofluorescent ring in the right eye and no abnormal hyperfluorescence in the left eye (Fig 4). She underwent a negative infectious work-up and was started on prednisone 50 mg daily with a slow taper.

One month later, fundus photos revealed complete resolution of the previously noted cream-colored lesions in both eyes. Following disease resolution, FAF showed hypoautofluorescent lesions with fewer reticulated areas of hyperautofluoresence in the right eye and no abnormal autofluorescence in the asymptomatic left eye. The absence of FAF findings in the left eye despite fundus photo and ICG evidence of disease suggested that the disease process was choroidal in nature, with secondary involvement of the outer retina and RPE, as evidenced by changes in the FA and FAF of the right eye. Specifically, FA demonstrated staining of the lesions in the right eye, while the angiogram was normal in the left eye. ICG showed areas of choroidal vascular dilation with irregular borders in the right eye without hypocyanescent lesions.

This patient was atypical in her older age at onset of the disease and absence of symptoms in the left eye at presentation. Given that the left eye did not have overlying RPE or photoreceptor disruption, this slightly asymmetric presentation at onset suggested that her disease was still evolving in the left eye at the time, and improved following corticosteroid treatment. Coxsackievirus B titers (CVB2 1:320; CVB4 1:80) were elevated during the course of her disease. She was tapered off prednisone over the next four months and maintained inactive disease without recurrence.

#### Patient 5

A 25-year old female was referred for the evaluation of new scotomas in the left eye without a viral prodrome. On initial evaluation, funduscopic examination revealed several pigmented areas of RPE scarring in the right eye and 8–10 cream-colored lesions in the posterior pole of the left eye (Fig 1). ICG confirmed intense hypocyanescent lesions that were visualized to a greater degree than could be appreciated clinically in the left eye. She underwent a negative infectious work-up and was started on prednisone 100 mg daily. Coxsackievirus titers at this initial visit revealed an elevated CVB2 titer (1:40) and an elevated CVB3 titer (1:160).

At the one-month follow-up visit, funduscopic examination showed complete resolution of cream-colored lesions in the left eye. Approximately one year later, the patient presented with a new scotoma in the left eye, and funduscopic examination revealed new cream-colored deep lesions near the fovea in the left eye (Fig 3). ICG showed multiple hypocyanescent lesions corresponding to the cream-colored lesions visualized on exam. High-dose prednisone (100 mg daily) was then initiated. CVB titers showed an increased coxsackievirus B3 titer (1:320) compared to the initial visit.

One month after disease recurrence, the patient’s examination revealed stable scarring in the right eye and increased hyperpigmentation overlying the previously active lesions in the left eye. Upon further discussion, the patient deferred starting immunosuppression and she was continued on a prednisone taper.

## Discussion

In this series of patients with APMPPE, multimodal diagnostic imaging was informative in identifying the structural changes that occur during the early phases of the disease and during disease resolution in long-term follow-up. Gass [1] first described APMPPE as a condition primarily affecting the RPE. Van Buskirk et al [27] and Deutman et al [28] felt the disease was most likely inflammatory with features consistent with a diffuse choriocapillaritis. In our study, the use of multimodal diagnostic imaging suggest that choroidal inflammation preceded the development of overlying RPE and photoreceptor damage. High-speed ICG angiography demonstrated dilated choroidal vasculature with well-circumscribed areas of hypocyanescence, which were potentially attributable to areas of non-perfusion or blockage from inflammation within the inner choroid or to blockage from overlying inflamed RPE. Lesions on FA, OCT, and FAF tended to be more limited and followed choroidal involvement overlying the areas of lesions detected by ICG, suggesting that either the RPE and outer retinal changes occur secondarily to the underlying choroidal process or that ICG is more sensitive in revealing RPE alteration than OCT and FAF. A similar finding was described in a study of ICG-A in 2 patients with APMPPE in which delayed choroidal filling and extensive choroidal non-perfusion were noted in early disease and recovered during disease resolution [16].

The anatomic changes were further characterized by OCT, which demonstrated significant disruption of the outer retinal architecture including the photoreceptor layer. This finding supports the notion that a choroidal vascular process may be an early event during disease evolution, as the outer retina receives its vascular supply via the choriocapillaris. Interestingly, the anatomic disruption in this layer appears to be partially reversible. SD-OCT findings from disease onset to resolution followed a characteristic pattern: choroidal thickening and vascular dilation, outer retinal disorganization with loss of the ELM and ellipsoid zone, partial restoration of the ellipsoid zone, and lastly, recovery of ellipsoid zone architecture in treated patients. Similar patterns of outer retinal disruption have been documented in previous reports [13].

FAF may also provide further insight into ongoing disease at the level of the RPE and outer retina. In early disease, discrete hypoautofluorescent lesions with surrounding diffuse hyperautofluorescence can be seen days after onset of symptoms, suggesting large areas of sick/swollen RPE cells. Over the course of weeks, these lesions appear more heterogenous in their autofluorescence, and as the disease resolves, mainly hypoautofluorescent lesions are observed. These lesions on FAF lagged behind the appearance of lesions on ICG-A. Over time as lesions heal, a confluent area of hypoautofluorescence can be observed signifying widespread RPE loss.

All patients underwent anti-inflammatory treatment for their disease: 1 received IV corticosteroid, all received oral corticosteroids, and 5 received additional corticosteroid-sparing immunomodulatory therapy. The dosages of oral corticosteroid were weight-based and patients with acute disease were treated with an initial dose of 1 mg/kg of prednisone. Corticosteroid taper was initiated after improvement of the lesions were observed. Intravenous corticosteroid was elected in one patient in whom there was concern for immediate, foveal-threatening disease. Currently, there are differing opinions in the literature regarding the need for therapy [12]. Given the association of APMPPE with a systemic, specifically cerebral vasculitis, risk of disease relapse and recurrence, and potential for severe vision loss, which can be associated with evolution to an ampiginous choroiditis or relentless placoid chorioretinitis phenotype, immunomodulation remains a strong consideration in APMPPE, particularly in patients with fovea-threatening lesions [29, 30]. Interestingly, in patient 3, the use of corticosteroids led to resolution of the choroidal lesions in the *asymptomatic* eye and it is possible that the early use of corticosteroid led to disease resolution and prevented her from developing RPE and photoreceptor damage that was observed in the symptomatic eye, which led to her initial ophthalmic evaluation.

The differential diagnosis of APMPPE includes other white dot syndromes such as serpiginous choroiditis, which should be considered particularly in chronic cases, Vogt-Koyanagi-Harada syndrome, and infectious entities including tuberculosis. Patient 4 had an extremely poor visual outcome with progressive vision loss despite aggressive immunosuppression. Furthermore, this patient was also diagnosed with mesenteric adenitis around the time of onset of her ocular symptoms, suggesting that an inflammatory process contributed to both ocular and systemic manifestations. Chronic disc edema, anterior/posterior segment inflammation, and recurrence suggested that she had more severe choroidal inflammation compared to the other patients in our study group, suggestive of atypical APMPPE, perhaps falling in the ‘ampiginous’ or relentless placoid chorioretinitis spectrum of disease. The mainstay of treatment for these patients with more severe pathology has been oral corticosteroids; however, corticosteroid-sparing immunosuppression (e.g. azathioprine, cyclosporine, and mycophenolate mofetil) has been used [31].

Coxsackievirus has previously been implicated in association with other ocular inflammatory conditions and posterior segment lesions [19–21, 32]. Kadrmas and colleagues described a case of chorioretinitis in a 34-year old woman with bilateral cream-colored parafoveal and mid-peripheral lesions, and discovered that CVB4 titers demonstrated a four-fold rise between the acute (1:4) and convalescent (1:64) sera [32]. Coxsackievirus B4 infection has also been associated with iridocyclitis and occlusive retinal vasculitis, with titers showing a 16-fold increase between the acute (1:8) and convalescent sera (1:128) [3]. There has been one prior report of an association between coxsackievirus and APMPPE that described a case of fulminant bilateral chorioretinitis and papillitis with elevated titers of CVB3, CVB4, and CVB5 [33].

Our study also raises the question of a potential association of coxsackievirus with APMPPE, as CVB titers were found to be elevated at a level ≥1:80 in all 6 patients who were tested in our series. Two of the patients demonstrated seroconversion with a four-fold increase in CVB titer between the acute and convalescent phase, suggesting concomitant acute infection or recent exposure at onset or recurrence of APMPPE. Disease recurrence was also associated with elevated CVB titers, as three patients showed an antibody titer of 1:320 during clinical recurrence of disease.

Multiple reports have further demonstrated an association between coxsackievirus and the development of unilateral acute idiopathic maculopathy (UAIM), a rare inflammatory condition that affects the choroid, retinal pigment epithelium, and outer photoreceptor complex in young adults [22, 23, 34, 35]. Beck and colleagues described elevated convalescent coxsackievirus A16 and B6 antibodies in association with UAIM, and felt that this disease entity should be renamed “coxsackievirus maculopathy” [23]. Positive coxsackievirus serotypes B1, B2, B5, and A9 have been reported in association with UAIM [22, 35]. In our case series of APMPPE patients, we found positive coxsackievirus serotypes B2, B3, B4, and B5 and elevated titers ≥1:80 in all patients that were tested for coxsackievirus. UAIM and APMPPE potentially may exist on a spectrum of inflammatory diseases, linked to coxsackievirus exposure in some individuals.

The potential mechanism of coxsackievirus injury in APMPPE and other inflammatory ocular disorders is unclear. One possible theory is that coxsackievirus injures tissue via direct viral infection. Coxsackievirus B5 was isolated in central nervous system (CNS) tissue in a case of choriomeningitis and has been hypothesized to grow in the choroid plexus gaining access to the CNS [36]. Furthermore, studies have shown that certain viruses including coxsackievirus B3 can infect RPE cells *in vitro*, and viral replication may lead to cytolysis [37]. A second theory involves an immune-mediated mechanism in which a transient viral infection triggers immune-mediated cell destruction. Similar to the mechanism of molecular mimicry in coxsackievirus-associated myocarditis, the expression of viral antigens may lead to upregulation of the immune system and a failure to distinguish between self (choroid, RPE) and non-self antigens (coxsackievirus) [38]. Hollsten and colleagues reported a case of coxsackievirus-associated APMPPE that demonstrated mild improvement with intravenous immunoglobulin (IVIG), supporting the likelihood of immune-mediated involvement [33]. Indeed, the favorable response to corticosteroids in our patients suggests that an immune-mediated mechanism may underlie the course of inflammation in APMPPE, and that coxsackievirus may constitute the trigger behind the inflammatory cascade.

Strengths of our study include the long-term follow-up and longitudinal analysis of multimodal imaging in this rare inflammatory syndrome. Additionally, coxsackievirus testing was obtained at multiple time points for many of these patients, allowing analysis of titer trends and correlation with the patients’ clinical courses. Limitations of the study include the small sample size and retrospective nature of this study. Additionally, a targeted polymerase chain reaction (PCR) test used to assess for viral infection is generally more sensitive when specific pathogens are suspected; however, there is a risk of false positive results due to the complexity of next generation sequencing methodologies [26]. Furthermore, it is possible that co-infection occurred in many cases, and other viruses were not tested.

In summary, multimodal imaging in combination with laboratory testing may be utilized in APMPPE to provide further insight into the pathophysiology and the clinical outcomes of disease. Although the etiology of this rare inflammatory disease is not well-characterized, coxsackievirus may play a role in triggering an immune-mediated mechanism associated with APMPPE. Analysis of multimodal imaging demonstrates that choroidal inflammation in APMPPE likely precedes RPE change and photoreceptor damage. Although there are no definitive guidelines about the role of immunosuppressive therapy in APMPPE, the risk of permanent vision loss, systemic vasculitis and recurrent disease warrants consideration of early therapy with corticosteroids. Further studies, however, are warranted to understand the underlying infectious and inflammatory triggers associated with APMPPE and potential therapeutic options.

## Supporting information

S1 Fig Fundus photos, fundus autofluorescence (FAF) and optical coherence tomography (OCT) images of APMPPE patients 1–3. Composite multimodal imaging show spectrum of choroidal and RPE lesions with hypo- and hyperautofluorescent patterns on FAF imaging. OCT images show varying patterns of outer retinal architectural change.
(TIFF)

S2 Fig Fundus photos, fundus autofluorescence (FAF) and optical coherence tomography (OCT) images of APMPPE patients 4–7. Composite multimodal imaging show spectrum of choroidal and RPE lesions with hypo- and hyperautofluorescent patterns on FAF imaging. OCT images show varying patterns of outer retinal architectural change. *FAF imaging was unavailable at this time point for patient 5.
(TIFF)

S1 File Minimal anonymized dataset. (PDF)
