# Supplementary figures and images for: Long-term multimodal imaging in acute posterior multifocal placoid pigment epitheliopathy and association with coxsackievirus exposure
Source: PLoS One. 2020 Aug 24;15(8):e0238080. doi: 10.1371/journal.pone.0238080 (PMC7446910; doi:10.1371/journal.pone.0238080)

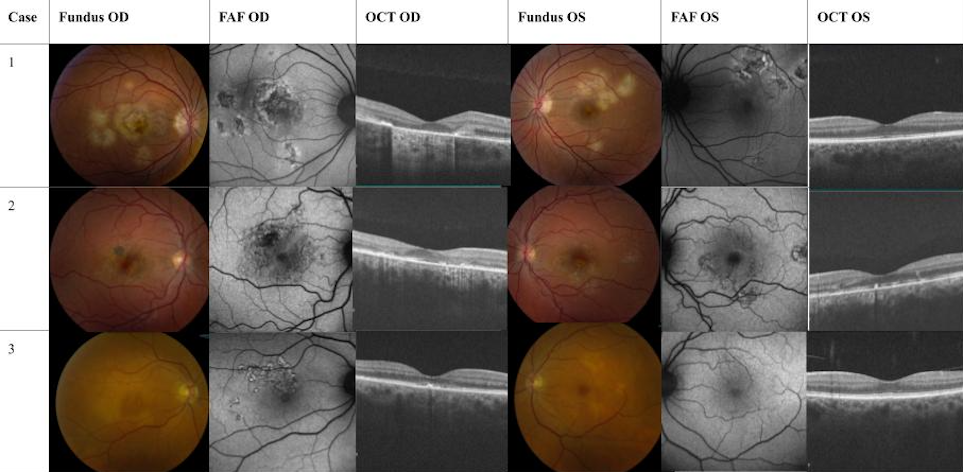

Supplement: S1 Fig — Composite multimodal imaging show spectrum of choroidal and RPE lesions with hypo- and hyperautofluorescent patterns on FAF imaging. OCT images show varying patterns of outer retinal architectural change. (TIFF) [file pone.0238080.s001.tiff]

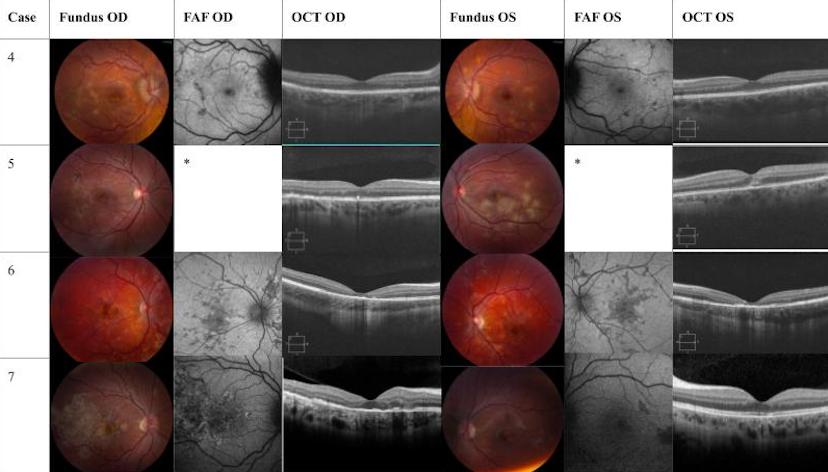

Supplement: S2 Fig — Composite multimodal imaging show spectrum of choroidal and RPE lesions with hypo- and hyperautofluorescent patterns on FAF imaging. OCT images show varying patterns of outer retinal architectural change. *FAF imaging was unavailable at this time point for patient 5. (TIFF) [file pone.0238080.s002.tiff]
