# Supplementary material for: Long-term multimodal imaging in acute posterior multifocal placoid pigment epitheliopathy and association with coxsackievirus exposure
Source: PLoS One. 2020 Aug 24;15(8):e0238080. doi: 10.1371/journal.pone.0238080 (PMC7446910; doi:10.1371/journal.pone.0238080)
Supplement: S1 File — (PDF) [file pone.0238080.s003.pdf]

Supporting Information 1 File : Minimal Anonymized Dataset

Patient #	Sex	Age (years)	Medical Condition	Viral Prodrome	Cerebral Vasculitis	Days from symptom onset to presentation	Initial Va (OD; OS; sx)	Final Va (OD; OS; sx)	FA	ICG-A	OCT Initial	OCT Final	FAF Initial	FAF Final	Treatment	CVB Acute titers	CVB Convalescent Titers	CVB Recurrence Titers	Total Follow-up (mo)
1	F	23	No	No	No	17	0.7; 0; central scotoma OD	0.1; 0; asx	Early blockage with staining and late leakage of lesions	Dilated choroidal vasculature; multiple choroidal perfusion defects/hypo-cyanescent lesions	CFT OD-212um OS-253um, disruption of outer retina	CFT OD-210um OS-248um, intact outer retina with mild RPE scar OD	Heterogeneous lesions with central HypoAF and surrounding HyperAF	HypoAF lesions	IV cortico-steroids with prolonged oral taper, MMF	B3 1:20; B4 1:160	B3 1:320; B4 1:160	N/A	9
2	F	25	No	No	No	180	0; 0; paracentral scotomas OU	0; 0; asx	N/A	N/A	CFT- OD - 210um OS-214um, disruption of outer retina	CFT OD-214um OS-218um, partial reorganization of outer retina	Heterogeneous lesions with central HypoAF and surrounding HyperAF	HypoAF lesions	Cyclosporine, Azathioprine, oral cortico-steroids	N/A	N/A	N/A	5
3	F	75	HTN	No	No	2	0.6; 0.3; inferior paracentral scotoma OD	0.18; 0.18; asx	Early blockage with late staining and leakage of lesions	Dilated choroidal vasculature, multiple choroidal perfusion defects/hypo-cyanescent lesions	CFT- OD-194um OS-245um, disruption of outer retina	CFT- OD 211um OS-243um, partial disruption of outer retina OD and mild ERM OS	HyperAF lesions surrounded by hypoAF ring OD, normal AF OS	HypoAF lesions with few stippled areas of hyperAF OD, normal AF OS	Oral cortico-steroids	N/A	B2 1:320; B4 1:80	N/A	34
4	F	16	Mesenteric Adenitis, Iron Deficiency Anemia	No	No	139	1.3; 0.8; central scotoma OU	1.18; 1; vision stable	Optic disc leakage and early blockage of lesions with late staining and leakage	Multiple choroidal perfusion defects/hypo-cyanescent lesions	CFT OD-273um OS-284um, disruption of outer retina OD, normal outer retina OS	CFT OD-248um OS-302um, intact outer retina OD, normal outer retina OS	Heterogeneous lesions with central HypoAF and surrounding HyperAF	HypoAF lesions	Oral cortico-steroids, retisert RSS OU, MMF, cyclosporine, MTX, azathioprine	N/A	N/A	B3 1:80; B5 1:80	85
5	F	25	No	No	No	42	0.3; 0.5; central scotoma OS	-0.12; -0.12; vision stable	Early blockage of lesions with late staining and leakage	Multiple choroidal perfusion defects/hypo-cyanescent lesions	CFT- OD-282um OS-332um, disruption in outer retina	CFT- OD-260um OS-305um, mild disruption in outer retina	N/A	HypoAF lesions OD, hypoAF lesions with few stippled areas of hyperAF OS	Oral cortico-steroids	B2 1:40; B3 1:160	N/A	B2 1:20; B3 1:320	20
6	M	14	No	Yes	No	74	0.88; 1; paracentral scotomas OU	0.18; 0.4; asx	Window defects, staining	Multiple hypo-cyanescent lesions	CFT- OD-178um OS-234um, disruption of outer retina	CFT- OD-282um OS-332um, disruption of outer retina	Heterogeneous lesions with central HypoAF and surrounding HyperAF	HypoAF lesions	Oral cortico-steroids, MTX	B3 1:80; B4 >=1:640	B3 1:20; B4 1:160	B3 1:80; B4 >=1:640	22
7	M	13	No	No	No	21	0.88; 0; central scotoma OD	0;0; asx	Early blockage and late staining	Multiple hypo-cyanescent areas	CFT- OD-224um OS-242um, disruption of outer retina	CFT- OD-178um OS-234um, partial reorganization of outer retina	Heterogeneous lesions with central HypoAF and surrounding HyperAF OD, hypoAF lesions OS	HypoAF lesions	Oral cortico-steroids, azathioprine, MMF	B3 1:320	B3 1:160	N/A	46

Legend: AF – autofluorescence; asx – asymptomatic; CFT – central foveal thickness; CVB – coxsackievirus B; ELM – external limiting membrane; ERM – epiretinal membrane; EZ – ellipsoid zone; F – female; FA; fluorescein angiogram; FAF – fundus autofluorescence; HTN – hypertension; hyperAF – hyperautofluorescence; hypoAF – hypoautofluorescence; ICG-A – indocyanine green angiography; IV – intravenous; M – male; MMF - mycophenolate mofetil; mo – month; MTX – methotrexate; N/A – not applicable; OCT – optical coherence tomography; OD – right eye; OS – left eye; pt – patient; RPE – retinal pigment epithelium; um – microns; Sx – symptoms; asx – asymptomatic Va – visual acuity
